# Self-Detection of the LH Surge in Urine After GnRH Agonist Trigger in IVF—How to Minimize Failure to Retrieve Oocytes

**DOI:** 10.3389/fendo.2020.00221

**Published:** 2020-04-21

**Authors:** Mauro Cozzolino, Sonia Matey, Abigail Alvarez, Mónica Toribio, Verónica López, Marta Perona, Elizabet Henzenn, Manuel Piró, Peter Humaidan, Juan A. Garcia-Velasco

**Affiliations:** ^1^IVI RMA Madrid, Madrid, Spain; ^2^Department of Obstetrics and Gynecology, Rey Juan Carlos University, Madrid, Spain; ^3^Department of Clinical Medicine, Aarhus and The Fertility Clinic Skive Regional Hospital, Aarhus University, Aarhus, Denmark

**Keywords:** LH surge, GnRH agonist, urinary test, trigger, oocyte retrieval

## Abstract

**Research question:** Urine LH testing may be useful to confirm an LH surge after the GnRH agonist (GnRHa) trigger prior to oocyte retrieval in IVF.

**Design:** A prospective cohort study, including oocyte donors undergoing ovarian stimulation, treated with a GnRHa trigger for final oocyte maturation. Urine LH testing was performed at home, 12 h after the GnRHa trigger. In the case of a negative result, serum LH and progesterone measurements were done that same day. Donors with no serum LH peak after trigger were re-scheduled using a dual trigger, with GnRHa and hCG.

**Results:** Three hundred and fifty nine oocyte donors were included in the analysis. Three hundred and fifty six donors had positive urine LH tests, followed by oocyte retrieval. In one case, the LH test was positive, however, no oocytes were retrieved (false positive 1/356). Three LH tests were negative in urine: in one of these three cases, LH was tested again in blood, confirming an LH rise, consistent with an optimal response to the GnRHa trigger; in the other two cases, serum LH was <15 mUI/mL, after which the oocyte retrieval was re-scheduled for 36 h after an being re-triggered, resulting in the retrieval of 19 and 22 MII oocytes, respectively. Considering the cost analysis, it would be a significantly cost-saving strategy, as blood testing would have costed 14,840€ vs. only 185.5€ in urine LH kits.

**Conclusions:** Urinary testing of the LH surge after GnRHa trigger is easy, safe, reliable, and convenient. In addition, LH urine testing allows identifying donors and patients who could benefit from a rescue hCG trigger after an unsuccessful GnRHa trigger.

## Introduction

In clinical practice, the total number of oocytes retrieved usually differs from the number of mature follicles observed on ultrasound as not all growing follicles will generate mature oocytes. Thus, it is not uncommon in IVF not to retrieve the same number of oocytes as mature follicles observed on ultrasound. However, only in a few cases, there is a complete failure to retrieve oocytes after hCG as well as the GnRHa trigger (1, 2). In the case of a GnRHa trigger, failure to retrieve mature oocytes should not always be classified as an Empty Follicle Syndrome (EFS) (3), as the pathophysiology is likely to be different. Based on the available literature, some authors do not support the existence of the EFS, considering the “syndrome” due to errors in the administration of the trigger. Moreover, EFS is defined as a sporadic event in patients with adequate ovarian stimulation and final oocyte maturation trigger, correctly administered (4). Failure to retrieve mature oocytes or cases with a high immaturity rate (2, 5) have been described in 1% of IVF oocyte retrievals (2, 6–8). Even though failure to retrieve oocytes after GnRH agonist triggering is relatively uncommon, there is a need to prevent the occurrence –a frustrating experience for both patients and physicians.

After the final oocyte maturation trigger with a bolus of GnRHa, the subsequent LH surge induced should determine the correct response of the patient to the subcutaneous GnRHa injection. Even more, it could allow the identification of patients in whom pituitary dysfunction might induce an insufficient endogenous LH surge, not enough to maturation of 70% of the oocytes. As a result of increasing worldwide use of GnRHa trigger for final oocyte maturation, primarily to avoid OHSS (9–11), the measurement of serum LH post-trigger has been suggested to prevent EFS. Unfortunately, a consensus on optimal serum LH threshold levels after the trigger has not yet been reached (12–14). Kummer et al. (8) detected low LH values as <15 mIU/ml 10 h after trigger in seven cases of EFS among 508 patients triggered with GnRHa. However, others used different thresholds (6). Interestingly, Shapiro et al. (15) demonstrated a dramatic reduction in the numbers and maturity rates of oocytes when serum LH values 12 h after the trigger was lower than 12 mUI/ml. After the GnRHa trigger, the subsequent LH peak lasting for ~28–32 h can be measured not only in serum but also in urine, using the same type of urine strips, that are currently used to detect the LH surge during the natural cycle (16).

In our oocyte donation program, the GnRHa trigger is routinely used, and during the years a few donor cycles resulted in an insufficient number of MII oocytes retrieved, even after a reassuring final ultrasound examination with good hormonal profile at the time of trigger. These cases are exceptional and unpredictable. Therefore, detecting the LH surge could be important to avoid failed oocyte retrieval in the event of error in the trigger administration, or an insufficient pituitary response to the GnRHa trigger. This prompted many clinics to recommend donors to have a serum measurement in the fertility clinic 12 h after the GnRHa trigger. However, urine LH measurement performed at home should predict the correct response to the GnRHa trigger, and if needed, the administration of a rescue bolus of hCG (re-trigger) and re-scheduling could avoid EFS or a low follicle mature oocyte ratio (17, 18).

The urinary LH test was firstly used in the context of intrauterine insemination (IUI), as 22% of patients exhibited a premature LH rise (19). In cycles where clomiphene citrate was used, up to 30% premature LH surges were reported, and up to 42% in rFSH cycles (20). Urinary LH testing helped to time the insemination in IUI cycles, thus, when it was performed between 18 and 53 h after a positive urine LH test, reasonable results in terms of live birth rate were obtained (21).

The present study aimed to explore whether the routine use of urine LH testing in oocyte donors 12 h after GnRHa trigger for final oocyte maturation would correctly detect an insufficient LH surge. Moreover, we have tried to identify patients that required a rescue hCG bolus to avoid failed oocyte retrieval.

## Materials and Methods

We performed a prospective cohort study, enrolling a total of 371 oocyte donors from May 2017 until November 2018. The study was approved by our Institutional Review Board (1701-MAD-006-JG) in compliance with the provision of the Spanish law on Assisted Reproductive Technologies (14/2006).

### Oocyte Donors

Oocyte donors were healthy women aged 18–35 years with regular menstrual cycles, no hereditary or chromosomal diseases, who had normal karyotype, body mass index (BMI) of 18–29 kg/m^2^, no more than two previous miscarriages, no gynecological or medical disorders, and a negative screening result for sexually transmitted diseases (19). Inclusion in the oocyte donor program also required the donor to have at least six antral follicles per ovary at the beginning of the cycle, and at least 8 follicles larger than 14 mm on the day of ovulation induction. Donor who had polycystic ovary syndrome based on the Rotterdam criteria (20) or sonographically visible endometriosis were excluded. Finally, donors were required to have a smartphone with WhatsApp (WhatsApp Ireland Limited, Dublin, Ireland) available to enter the trial.

### Ovarian Stimulation

For planning purposes, an oral contraceptive pill (Microgynon®, levonorgestrel 0.15 mg/ethinylestradiol 0.03 mg, Bayer Hispania, Spain) was administered between 12 and 16 days prior to ovarian stimulation, starting on day 1 or 2 of the menses of the previous cycle. Following a 5-day wash-out period after the last pill, donors started the ovarian stimulation protocol. Donors received daily doses of 150/225 IU of recFSH (Gonal-f® Merck Serono, Spain) depending on body mass index (BMI) and antral follicle count. A daily dose of 0.25 mg GnRH antagonist (Cetrotide® Merck Serono, Spain) was introduced when one follicle reached a mean diameter of 13 mm. A single dose of 0.2 mg GnRHa (Decapeptyl® Ipsen Pharma, Spain) was administered to trigger final oocyte maturation when at least three follicles reached a mean diameter of 17–18 mm. The triggering bolus of GnRHa was self-administered by the donor. To avoid failure to comply prior to treatment all donors received training in a practical nurse-led workshop related to drug preparation and administration; moreover, donors received instructions as to how to download a video available on the clinic's website with a step-by-step drug instruction. As part of the general practice of the clinic, oocyte pick-up was performed under sedation 36 h after GnRHa trigger.

### Urine LH Testing

The LH test was performed at home in urine, deriving from the first micturition in the morning, 12 h after the GnRHa trigger. Donors were instructed how to self-administer the GnRHa trigger bolus 36 h prior to oocyte retrieval and to perform the urine LH test the following morning during the first micturition. All donors received the LH urine test from the clinic (LH test, Lab Ruedafarma, Spain). With the instructions, donors were also provided with an emergency phone number for further instructions if they encountered any problems.

The lower sensitivity of the urine LH test mentioned is 25 mIU/mL. To perform the test, donors had to collect the urine in a plastic container and then remove the strip from the protective wrapper. The strip was immersed in the urine sample for 5 s, after which it was removed and placed on a clean, dry, non-absorbent surface. Importantly, donors had previously been thoroughly instructed on the possible outcomes of the test; specifically, in case of a positive result, resulting in two purple lines on the strip. The results were available within 40 s to 10 min.

### Pictures of LH Tests

Donors were instructed to send a cell phone picture of the LH test to the nurse via WhatsApp (WhatsApp Ireland Limited, Dublin, Ireland) application for smartphone as soon as the test was performed. In the case of a positive LH test, donors underwent oocyte retrieval the following morning as scheduled. In case of a negative result, the donor was asked to visit the clinic the same day to have a serum LH and progesterone measurement. To evaluate the validity of the test, the false positive rate was calculated by false positive/(true negative + false positive), and the false-negative by false negative/ (true positive +false negative).

### Serum LH Analyses in LH Negative Urine Tested Patients

Serum LH levels were analyzed in patients with a negative urine test only, using an automated electro-chemiluminescent immunoassay system (Roche Cobas e411 analyzer). The sensitivity for LH was 0.1 mIU/ml. Donors with no serum LH surge defined as LH <15 mIU/ml (12) at 12 h after injection and with more than 19 ovarian follicles >11 mm were given in the clinic by a nurse a dual trigger consisting of a bolus of GnRHa (0.2 ml Decapeptyl) and hCG, 1000 IU (Ovitrelle, Merck Serono) in order to minimize the risk of OHSS development; oocyte retrieval was subsequently performed 36 h after re-trigger. In donors with <19 follicles >11 mm, hCG 6500 UI (Ovitrelle, Merck Serono) was administered. Patients with a negative urine test, regardless of their serum LH levels, continued with GnRH antagonist the day of the serum LH testing until the new trigger was administered.

### Cost Analysis

The cost analysis was performed from a patient perspective and was limited to costs from LH testing to oocyte retrieval. Direct non-medical costs (travel costs) data were based on a previous review (22). The theoretical cost if every patient had come in for serum LH testing, including indirect costs such a travel time and income loss was compared against the cost of a home urine LH kit. For the calculations, we considered serum LH cost 30€ and the urine LH kit cost 0,5€.

## Results

**Figure 1:**
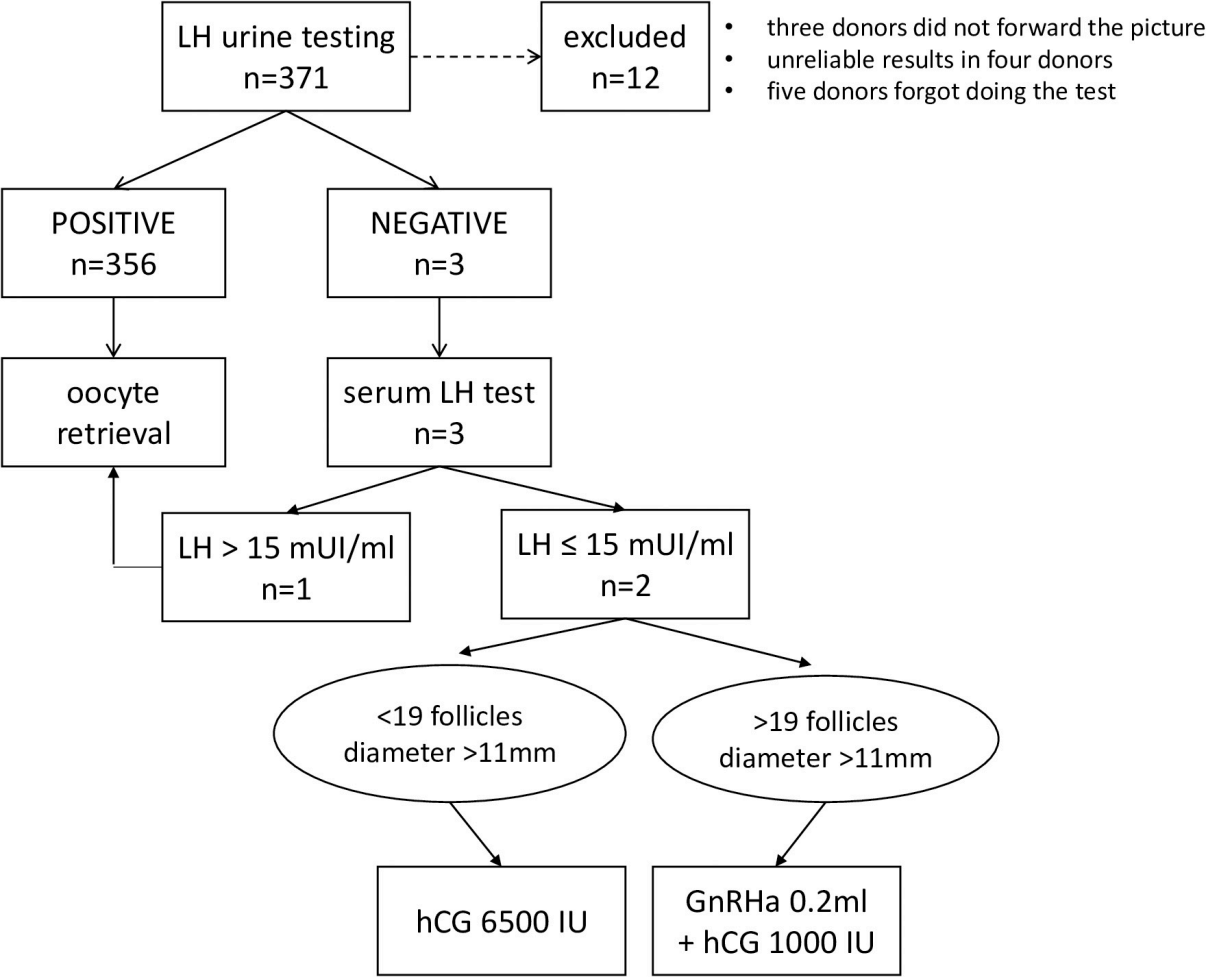
Flow chart according to LH urine testing results.

Three hundred and seventy one donors were enrolled in the study, and 12 donors were excluded as they did not perform urine LH testing as instructed, leaving 359 donors included in the analysis (Table 1). Three donors did not forward the picture to confirm the test results, in four donors the result was unreliable due to the lack of a control line to confirm the correct execution of the LH urine test, and five donors forgot to perform the urine test (Figure 1).

**Table 1:** General demographics of the oocyte donor population included in the study.

Patients	359
Age (years)	25.9 ± 1.4
BMI (kg/m^2^)	23.1 ± 1.2
Antral follicular count	18.3 ± 5.1
Duration of stimulation (days)	12.1 ± 1.4
Total gonadotrophin dose (IU)	1,861 ± 689
Estradiol (pg/mL)	3,105 ± 664
Number of oocytes retrieved	19.2 ± 4.9
Number of MII oocyte	13.7 ± 2.6

Finally, a total of 359 oocyte donors and 359 oocyte retrievals were included in the present analysis. 356 donors (99.16 %) had a positive urine LH test prior to oocyte retrieval. In one donor, the urine LH test was positive (1/356, 0.28 % false-negative rate), however, no oocytes were retrieved. Three urine LH tests were negative. One was false-negative in fact LH was tested in blood the same morning, confirming an LH rise corresponding to 18.6 mUI/mL consistent with an adequate response to the GnRHa trigger bolus. In the other two cases, after the negative urine LH test, serum LH testing in the clinic revealed a serum LH level <15 mUI/mL, resulting in re-scheduling with an hCG trigger, oocyte retrieval after 36 h and the retrieval of 19 and 22 MII oocytes, respectively. Table 2 summarizes the general characteristics of the four oocyte donors who experienced either false positive or false negative urine LH testing. The false-positive rate was 33% (1/3) and the false-negative rate was 0.28% (1/356), with a sensitivity of 99.7% (355/356), and specificity of 66.7% (2/3) (Table 3). Regarding the cost analysis, and considering the cost of the urine LH kit (0,5€ per sample) and the cost of serum LH testing (30€ per sample) in addition to an estimate of the cost of visiting the clinic (10€ round trip), it would be a significantly cost-saving strategy, as blood testing would have costed 14,840€ vs. only 185,5€ in urine LH kits.

**Table 2:** General characteristics of the patients with abnormal results in the urine LH test or failed oocyte retrieval after a positive LH test in urine.

**Patients**	**BMI (kg/m^**2**^)**	**Duration of stimulation (days)**	**Total gonadotrophin dose (IU)**	**Urine LH test**	**Serum LH (mIU/mL)**	**Progesterone (ng/mL)**	**Estradiol (pg/mL)**	**N**°**Follicles>11 mm**	**Re-trigger**	**# oocytes retrieved**	**# MII**
1	20.2	10	1,500	+−	18.6	27	1,280	27		22	18
2	28.2	11	2,225	—	7.3	14	1,403	25	hCG+GnRHa	24	19
3	25.3	13	2,925	+++			2,380	20		0	0
4	21.4	11	2,000	—	12.1	20.2	3,423	36	hCG+GnRHa	32	22

*+++, positive; −, negative; +−, slightly positive*.

**Table 3:** Summary of parameters of urinary LH test: true-positive rate (sensitivity) 355/356 = 99.7%; false-positive rates (1-specificity) 1/3 = 33.3%; false-negative rate (type 2 error) 1/356 = 0.28%; specificity 2/3 = 66.7%.

	**Serum LH>15 IU/l (or oocytes retrieval uneventful)**	**Serum LH <15 IU/L (or no oocytes retrived)**	
Urine LH	TP	FP	356
+	355	1	
Urine LH	FN	TN	3
–	1	2	
	356	3	359

*TN, true negative; FN, false negative; FP, false positive; TP, true positive*.

## Discussion

In this prospective cohort study performed in a total of 359 oocyte donors triggered with a bolus of GnRHa for final oocyte maturation, was explored the predictability of urine LH self-testing 12 h after trigger to avoid failure of oocyte retrieval. In 356 patients (99%) the LH urine assay was positive, leading to oocyte retrieval with normal mature oocyte ratio. One patient had a false-positive result, with a positive urinary LH test but no oocytes were retrieved. In three patients LH testing in urine was negative, however, only two patients had serum LH levels requiring re-trigger and re-scheduling.

This new approach of self-testing of the LH surge in urine and communication/confirmation via cell phone photo provides a simple, safe, convenient, and economical method to confirm that the LH surge was adequately induced by the GnRHa trigger. This model could be used in all patients undergoing oocyte retrieval after a GnRHa trigger, helping to detect errors in the administration and/or inadequate responses to the trigger, improving patient compliance and minimizing failure to retrieve oocytes in IVF.

The use of GnRHa for triggering of final oocyte maturation is the new paradigm as a safer alternative to hCG to prevent OHSS. The mechanism by which the GnRHa trigger is effective seems to be dependent solely on the resulting LH activity (12). After the administration of GnRHa, the immediate LH surge is followed by receptor down-regulation (23). Although data has demonstrated efficacy and safety of GnRHa trigger, the administration may not always result in an adequate oocyte yield in a subset of patients (6, 8, 12, 14). As a consequence of an inadequate action, fewer oocytes may be retrieved than expected from the number of mature follicles visible on the day of triggering (14). Failure to retrieve oocytes is a rare and frustrating event that results in cycle cancellation. The complete failure to retrieve oocytes when final oocyte maturation trigger is administered correctly –either using GnRHa or hCG- is considered a “genuine” EFS, whereas “false” EFS is a condition of failed oocyte retrieval when the trigger was not administered correctly (2, 9). Although the exact origin of the failure to retrieve oocytes remains elusive, dysfunctional foliculogénesis in older patients has been suggested as a cause, probably secondary to altered apoptotic inhibition of the granulosa cell (24). Furthermore, the age of the patient has been reported to be an important factor to increase the recurrence of EFS after hCG trigger, considering that between the age of 35–39 years the recurrence rate was 24%, reaching 57% for patients above 40 years (25). The debate on the causes of retrieval failure in the young population, excluding errors of administration, remains open. However, among the possible causes, molecular mechanisms underlying slow or insufficient follicular cell response to the LH stimulus is not yet understood. However, it could involve LH receptor polymorphisms, the presence of variant LHβ or the in-efficiency of post-receptor signal-transduction pathways (2).

In young patients triggered with GnRHa, a 'borderline' hypothalamic-pituitary dysfunction with levels of gonadotropins above the hypogonadotropic/hypogonadal level was hypothesized (2, 8). A temporary state of hyposensitivity of the pituitary gland could also explain EFS. Low LH release after the trigger in borderline hypogonadotropic/hypogonadal patients would allow follicular development, and initial luteinization of the follicle; however, it would be insufficient for the completion of cumulus expansion and detachment from the follicular wall (6).

Interestingly, Christopoulos et al. (17) reported six EFS patients after GnRHa trigger, having a mean age of 32.6 years, a mean antral follicle count of 27 and a mean basal FSH level of 4.4 UI/l, rather far from the LH value defining the hypogonadotropic hypogonadal patient.

Although the reasons for failed oocyte retrieval in healthy and older patients may be different, the diagnosis and treatment are still the same. Urinary LH test could be helpful for patients undergoing IVF treatment using GnRHa trigger. The use of urine LH test in oocyte donors has several important advantages, allowing to mitigate the risk of failure at oocyte retrieval, making the whole donation process more efficient, eliminating unnecessary costs for the clinic, avoiding blood testing in the clinic, and reducing psychological stress from cycle cancellation, not only for the donor but also for the recipient.

The strategy to schedule a re-trigger after failed oocyte aspiration in six or seven mature follicles has been proposed as a more efficient and cost-effective strategy compared to laboratory analysis alone (26). Rather than routine screening strategy, maybe we could consider identifying risk factors –such as low initial LH levels suggesting hypothalamic hypofunction- for an inadequate response as more useful in a clinical setting. However, this strategy might increase the costs, unless hormonal screening is performed routinely at the initiation of an IVF cycle, as in some academic programs. There are two limiting factors for this novel approach: firstly, urine LH test have not the same sensitivity, so universal application should be suggested with caution; secondly, the need to rely on patients performing the urine test themselves, which leaves room for human error, emphasizing the need of training. However, our approach in which the result of the test was confirmed by a trained nurse via a cell phone picture reduces the risk of error.

Given the low number of patients who did not respond with an LH surge (*n* = 3), the specificity of testing was understandably low at 66.7%, but the sensitivity was 99.7%. Urinary LH test could be a optimal test to confirm the LH surge in those patients triggered with GnRHa, and reasonably good for those patients without LH surge after triggering. Considering the low incidence of failed LH surges after GnRHa trigger, probably testing in blood might be more precise, but the inconvenience of traveling to the clinic one more day during the treatment cycle in addition to the costs make urine LH self-testing a very cost-effective test. In fact, as shown, it would be a significant cost-saving strategy, as blood testing would have costed 14,840€ vs. only 185,50€ in urine LH kits. Also, the strategy to schedule a re-trigger after failed oocyte aspiration once six or seven mature follicles have been aspirated could be interesting in terms of cost savings. However, patients going by through reiterated oocyte retrievals are subjected to psychological and emotional stress.

The main limitation of our study is that it was conducted in oocyte donors, most of whom presumably have a well-functioning pituitary-ovarian axis. It is unknown whether these results would translate to older women in general IVF practice. Also, the lack of a control group where no testing was performed represented a limitation of the study, but difficult to justify in a real-life clinical setting.

The use of a home urinary LH assay kit to detect ovulation in a GnRHa triggered cycle has several advantages over blood LH testing. Although IVF has increased worldwide, access to fertility care remains restricted for many patients. Increased attention to developing low-cost, effective, and accessible treatment alternatives must be prioritized (27). In this sense, the use of urine LH assay could reduce the stress and costs of fertility care program (27). In a randomized controlled study in patients undergoing donor IUI that used urinary LH assay, was observed a significant reduction in the number of visits to the clinic per cycle, without a significant impact on monthly fecundity rate or cumulative conception rates compared to serum LH assay (28). Also, the use of a urinary LH test could detect patients without LH surge after a GnRHa trigger that could benefit from a “rescue trigger” with a low-dose hCG.

In conclusion, this study validated the efficacy of LH urine surge testing after the GnRHa trigger in oocyte donors. A total of 99% of donors had a positive LH urine surge 12 h after the trigger, resulting in good oocyte retrieval rates, and, importantly, false positive and false negative rates of urine testing were very low; importantly, this novel concept used after GnRHa trigger allowed the identification of donors who would benefit from a re-trigger. Urinary LH surge testing is easy, safe, convenient, and reliable. Thus, this approach may constitute an alternative for clinicians and patients to minimize the failure of oocyte retrieval. Patients can safely perform the test at home, take a cell phone picture of the stick and communicate directly with the clinic without having to spend time for a blood sampling. Only in the few patients with a negative result in urine, an additional blood sample is needed to plan the continued handling of the patient; thus, this concept fulfills the demands of modern IVF in terms of safety, efficacy and patient friendliness.

## Data Availability Statement

The datasets analyzed in this article are not publicly available because the data contains identifiable information. Requests to access the datasets should be directed to Dr. Mauro Cozzolino, mauro.cozzolino@ivirma.com.

## Ethics Statement

The studies involving human participants were reviewed and approved by Hospital Puerta de Hierro. The patients/participants provided their written informed consent to participate in this study.

## Author Contributions

MC wrote the first and final drafts of the manuscript, performed the statistical analysis, interpreted results, and the literature search. SM, AA, MT, VL, and MPe were responsible of data setting and result collection. EH and MPi recruit the patients included in the study. PH designed the study and critically revised the manuscript. JG-V designed the study, interpreted results and critically revised the manuscript in the first and final drafts.

## Conflict of Interest

The authors declare that the research was conducted in the absence of any commercial or financial relationships that could be construed as a potential conflict of interest. The handling Editor declared a past co-authorship with the author PH.
